# Prevalencia de trastornos del gusto en niños y adolescentes con infección por coronavirus: revisión sistemática

**DOI:** 10.21142/2523-2754-0902-2021-061

**Published:** 2021-06-21

**Authors:** Cesar Andrés Borja-Villanueva, Luis Alexis Bernuy-Torres, Ivonne del Rocío Hernández-Romero, Zaré Huayaney-Velarde, Erika Ruth Alvarado-Muñoz

**Affiliations:** 1 Universidad Privada Juan Pablo II. Lima, Perú. abv1979@gmail.com, rociohero@gmail.com Universidad Juan Pablo II Universidad Privada Juan Pablo II Lima Peru abv1979@gmail.com, rociohero@gmail.com; 2 Universidad Nacional Mayor de San Marcos. Lima, Perú. luisbernuytorres@gmail.com Universidad Nacional Mayor de San Marcos Universidad Nacional Mayor de San Marcos Lima Peru luisbernuytorres@gmail.com; 3 Universidad San Martín de Porres. Lima, Perú. zare_huayney@usmp.pe Universidad de San Martín de Porres Universidad San Martín de Porres zare_huayney@usmp.pe Lima Peru; 4 Universidad Científica del Sur. Lima, Perú. ealvarado@cientifica.edu.pe Universidad Científica del Sur Universidad Científica del Sur Lima Peru ealvarado@cientifica.edu.pe

**Keywords:** trastornos del gusto, infecciones por coronavirus, ageusia, disgeusia, niños, adolescentes, taste disorders, coronavirus infections, ageusia, dysgeusia, children, teenagers

## Abstract

**Objetivo:**

Determinar la prevalencia de trastornos del gusto en pacientes niños y adolescentes diagnosticados con infección por coronavirus, a través de la evidencia en la literatura científica.

**Materiales y métodos:**

Una revisión sistemática de los artículos publicados entre el 19 de diciembre del 2019 y el 20 de diciembre del 2020 en las bases de datos Medline, Lilacs, BVS, Cochrane, SCOPUS y ScienceDirect. La estrategia de búsqueda de información se basó en el diagrama de flujo clásico de PRISMA. Para la evaluación del riesgo de sesgo, se usó la escala Newcastle-Ottawa.

**Resultados:**

Se encontraron 443 artículos en seis bases de datos y se incluyó un total de 7 artículos después de la evaluación, según los criterios de selección. Los artículos abordaron la variable de trastornos del gusto en tres formas: ageusia, disgeusia e hipogeusia, y se halló que esta manifestación clínica estaba presente desde los inicios de la infección.

**Conclusiones:**

Se ha encontrado una prevalencia de trastornos del gusto en niños y adolescentes diagnosticados con infección por coronavirus desde un 3,3% hasta un 26,9%.

## Abstract (en)

**Aim:**

To assess the prevalence of taste disorders in children and adolescents diagnosed with coronavirus infection according to the evidence reported in the scientific literature.

**Materials and Methods:**

A systematic review of articles published between December 19, 2019, and December 20, 2020 in the Medline, Lilacs, BVS, Cochrane, SCOPUS and ScienceDirect databases. The information search strategy was based on the classic PRISMA flow diagram. The Newcastle-Ottawa scale was used to assess the risk of bias.

**Results:**

443 articles were found in six databases, and a total of 7 articles were included after evaluation according to the selection criteria. The articles addressed the variable of taste disorders in three ways: ageusia, dysgeusia and hypogeusia; finding that this clinical manifestation was present from the beginning of the infection.

**Conclusions:**

The prevalence of taste disorders in children and adolescents diagnosed with coronavirus infection is from 3.3% to 26.9%.

## INTRODUCCIÓN

La enfermedad por coronavirus 2019 (COVID-19), causada por el SARS-CoV-2, se reportó por primera vez en Wuhan, China, en diciembre de 2019 y fue declarada pandemia por la Organización Mundial de la Salud (OMS) el 11 de marzo del 2020 ^1^. En los inicios de la pandemia, fueron la fiebre, la tos seca y el cansancio los síntomas asociados con una infección por COVID-19 ^2^^,^^3^, pero la historia natural de esta enfermedad se encuentra en permanente evolución y aún no se conocen con exactitud todas sus manifestaciones clínicas. Los Gobiernos y sus equipos de salud han ido informando sus datos clínicos y epidemiológicos, y se han incorporado nuevos signos y síntomas como los cardiovasculares, neurológicos, respiratorios, gastrointestinales, cutáneos y sensoriales ^4^^,^^5^.

En los países que empezaron con la política de rastreo del virus de forma masiva y el cerco epidemiológico, se reportó la aparición de trastornos del gusto y el olfato incluso antes de la confirmación por prueba molecular ^6^^-^^8^. Estos pacientes no presentaban ningún síntoma como fiebre o tos. Por ello, la Academia Estadounidense de Otorrinolaringología - Fundación de Cirugía de Cabeza y Cuello, la Sociedad de Otorrinolaringología del Reino Unido (ENT UK) y la Sociedad de Otorrinolaringología del Uruguay tuvieron a bien recomendar que apenas se presentan los trastornos del gusto y el olfato deben aislarse e informar a los servidores de salud ^9^^-^^11^. En el caso de los pacientes niños y adolescentes, la prevalencia de la enfermedad por coronavirus 2019 no está del todo clara, pues algunos reportan que va del 1% al 5% de todos los casos de COVID-19 ^12^, mientras que otros reportan el 12,3% ^13^ y en el Perú se ha reportado que el 40% de niños y adolescentes ya estarían infectados ^14^. Los síntomas más comunes en niños con COVID-19 confirmado y presunto son fiebre, tos, diarrea y dolor abdominal ^(15, 16)^ también se presentan los trastornos del gusto en 3 formas: hipogeusia (pérdida parcial del gusto), ageusia (pérdida total del gusto) y la disgeusia (gustos distorsionados) ^17^.

El presente trabajo consiste en una revisión sistemática interesada en responder la pregunta de investigación: ¿Cuál es la prevalencia de trastornos del gusto en niños y adolescentes diagnosticados con infección por coronavirus? 

Por ende, el objetivo del estudio es determinar la prevalencia de trastornos del gusto en pacientes niños y adolescentes diagnosticados con infección por coronavirus, para identificar la evidencia en la literatura científica.

## MATERIALES Y MÉTODOS

### Fuentes de datos y estrategia de búsqueda de información

La búsqueda de la información la realizaron dos investigadores sin conflicto de interés alguno basándose en el diagrama de flujo clásico de PRISMA. La pregunta de investigación se construyó con la estrategia PICO (P: niños y adolescentes diagnosticados con infección por coronavirus, I: trastornos del gusto, C: no se utilizó una comparación, O: determinar la prevalencia de los trastornos del gusto). Se utilizaron las bases de datos Medline, BVS/Lilacs, Cochrane, SCOPUS, ScienceDirect y ProQuest, las cuales fueron consultadas entre el 20 y el 27 de diciembre del 2020 (Tabla 1). Para la gestión documental de la información se utilizó el software Mendeley con el objetivo de identificar todos los artículos por duplicado, promover una mayor fiabilidad de selección y proceder al paso de criterios de elegibilidad. Las investigaciones abordan el problema de los trastornos del gusto a partir de su clasificación en hipogeusia, ageusia y disgeusia; gran parte de estos trabajos reportaron los trastornos del gusto asociados a los trastornos del olfato.

**Tabla 1:** Artículos encontrados por base de datos

Base de datos	N.º de artículos
Medline	63
BVS/Lilacs	26
Scopus	10
Cochrane	0
ScienceDirect	78
ProQuest	253
Total	430

Fuente: Elaboración propia

La estrategia de búsqueda utilizó los términos (DeCS/MeSH) y palabras como “taste disorders”, “ageusia”, “disgeusia e hipogeusia”, “coronavirus infections”, “pediatric”, “teen”, “child COVID-19” en diferentes combinaciones.

### Criterios de selección y elegibilidad de estudios (criterios de inclusión y exclusión)

**Figura 1:**
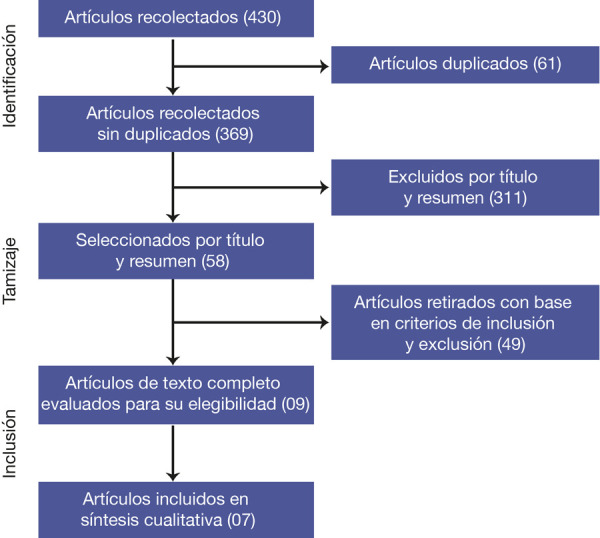
Flujograma de selección de fuentes primarias

La inclusión de los artículos fue producto de un proceso que se muestra en el diagrama de flujo típico para revisiones sistemáticas (Figura 1). Primero, se excluyeron los trabajos duplicados; luego de ello, los títulos y resúmenes fueron revisados al detalle y solo se incluyó a los que presentaban datos sobre los trastornos del gusto. Posteriormente, se leyó cada artículo para identificar completamente los datos relevantes. Solo se revisaron y analizaron los estudios que cumplieron con los criterios de inclusión. 

Se incluyeron estudios que investigaron la prevalencia e incidencia de los trastornos del gusto en pacientes niños y adolescentes con diagnóstico de COVID-19. Los artículos debían estar publicados entre el 19 de diciembre del 2019 y el 20 de diciembre del 2020, en cualquier idioma, y ser estudios observacionales a texto completo; por el contrario, se excluyeron los trabajos que no correspondan a estudios observacionales como cartas al editor, actas de congresos, editoriales, estudios en animales, artículos de revisión (sistemática o narrativa) y metaanálisis.

### Evaluación del riesgo de sesgo

Dos revisores por separado analizaron el texto completo de los estudios. El riesgo de sesgo se evaluó con la escala Newcastle-Ottawa para estudios observacionales ^18^^,^^19^. La evaluación fue realizada por dos investigadores y los resultados obtenidos fueron producto del consenso. La puntuación máxima posible fue de 10 puntos (0-4: calidad insatisfactoria, 5-6: calidad satisfactoria, 7-8: calidad buena y 9-10: calidad muy buena). Como se puede ver en la Tabla 2, se evaluaron los siete estudios incluidos para la revisión sistemática y se obtuvo que dos artículos alcanzaron la calidad satisfactoria y cinco artículos, calidad buena.

**Tabla 2:** Características y resultados de la evaluación del riesgo de sesgo para los estudios incluidos

Primer autor	Representatividad de la muestra	Usuarios seleccionados	Tamaño de la muestra	Determinación de la exposición	Factores de confusión controlados	Determinación del método	Análisis estadístico	Evaluación final
Krajcar N *et al.*^21^	0	1	0	2	2	1	1	7
Gorelov A *et al*. ^22^	0	1	0	2	2	1	0	6
Picao De Carvalho C *et al.*^23^	0	1	0	2	2	1	0	6
Ozores A *et al.*^24^	0	1	0	2	2	1	1	7
Chua G *et al.*^25^	0	1	0	2	2	1	1	7
Qiu C *et al.*^26^	0	1	0	2	2	1	1	7
King J *et al.*^27^	0	1	0	2	2	1	1	7

Fuente: Elaboración propia

Todos los estudios realizaron una selección no aleatoria y por conveniencia de la muestra, lo que compromete su representatividad; sin embargo, 4 de ellos tuvieron acceso a un marco muestral comprehensivo de la variable estudiada (Registro Nacional de Enfermedades Infecciosas de Croacia, Informes del Servicio Federal de Vigilancia de los Derechos del Consumidor y Bienestar Humano, Plataforma COVID-Hospital Universitario de Lisboa e Informe Salud Pública de Alberta), lo que mejora su posición con respecto a la selección de las unidades de análisis. La selección de los participantes tuvo criterios de inclusión y exclusión definidos previamente, con lo que se controlaron factores que pudieran influir en los resultados.

Los tamaños de las muestras estudiadas no se calcularon con fórmulas estadísticas. En cuanto a la determinación de la exposición, se usaron registros clínicos, bases de datos internacionales, registros hospitalarios, y se combinaron con entrevistas estructuradas a la muestra. En cuantos los factores de confusión, estos se encontraban controlados dentro de cada trabajo. La evaluación de los resultados se realizó en todos los estudios mediante un informe específico y los métodos fueron descritos y validados en todos los estudios. En cuanto a las pruebas estadísticas, se realizaron pruebas descriptivas con frecuencias absolutas y relativas para la variable estudiada.

## RESULTADOS

Se encontraron 443 artículos en seis bases de datos, se eliminaron 74 estudios por duplicado y se seleccionaron 369 para la lectura de títulos y resúmenes, de los cuales se excluyeron 311 por ser intrascendentes para la revisión (algunos evaluaban la variable en otro grupo etario y otros no aportaban resultados de prevalencia). De los 58 estudios seleccionados se excluyeron 49 según los criterios de inclusión y exclusión (diferente diseño del estudio, diferente tipo de artículo como revisiones sistemáticas, metaanálisis, revisiones narrativas y no incluyeron los textos completos), de los 9 artículos a texto completo se evaluó su elegibilidad y se excluyeron 2 por no aportar datos trascedentes para la revisión sistemática, por lo que se incluyeron un total de 7 artículos en el análisis cualitativo. Todo el proceso se describe en el cuadro 1 que evidencia el diagrama de flujo seguido en la presente revisión sistemática.

Para la presentación de los resultados, se creó un formulario estándar basado en el modelo de Cochrane ^20^ y se consideraron las principales características de los artículos seleccionados: título, autores, ubicación del estudio, diseño metodológico, tamaño de la muestra, edad de la muestra, el método de diagnóstico de la infección por coronavirus y el método de la evaluación de los trastornos del gusto. Estos datos se presentan en las Tablas 3 y 4.

**Tabla 3:** Estudios seleccionados para la síntesis cualitativa

Artículo	Título	Autor	Diseño del estudio	Año de publicación
1	Características epidemiológicas y clínicas de los niños y adolescentes croatas con enfermedad por coronavirus 2019 confirmada por examen de PCR: diferencias entre la primera y segunda ola	Krajcar N *et al*. ^21^	Observacional	2020
2	Infección por coronavirus COVID-19 en niños de la Federación de Rusia	Gorelov A *et al*. ^22^	Observacional	2020
3	Serie de casos de 103 niños con infección SARS-CoV-2 en Portugal	Picao de Carvalho C *et al*. ^23^	Observacional	2020
4	Clínica diferencial en niños infectados por SARS-COV-2, trazabilidad de contactos y rentabilidad de pruebas diagnósticas: estudio observacional transversal	Ozores A *et al.*^24^	Observacional	2020
5	COVID-19 en niños de tres regiones asiáticas cosmopolitas	Chua G *et al*. ^25^	Observacional	2020
6	Disfunción olfativa y gustativa como un identificador temprano de COVID-19 en adultos y los niños: Un estudio multicéntrico internacional	Qiu C *et al*. ^26^	Observacional	2020
7	Síntomas asociados con un resultado positivo de un hisopo para la infección por SARS-CoV-2 en niños de Alberta	King J *et al*. ^27^	Observacional	2020

Fuente: Elaboración propia

**Tabla 4:** Características de los datos obtenidos de los artículos incluidos en la síntesis cualitativa

Artículo	Autor	N:n%	Edad	Etnia n (%)	Diagnóstico de COVID-19	Método de evaluación del gusto	Prevalencia n (%)
1	Krajcar N *et al*.	151: (no diferenciaron sexos)	10.0 +/- 4.6	europea (100)	PCR	Análisis de las historias clínicas y entrevistas con los padres	27(17,9)
2	Gorelov A *et al*.	7649: (no diferenciaron sexos)	8,9 +/- 0,08	Rusa (100)	PCR	Análisis de las historias clínicas	1277(17,1)
3	Picao de Carvalho C *et al*.	103 (no diferenciaron sexos)	No se tiene registro	europea (100)	PCR	Análisis de las historias clínicas electrónicas y físicas, encuesta telefónica con tutores	4(4)
4	Ozores A *et al*.	33: 22(66,7%)M; 11(33,3%)	8,4 +/- (6,8-10,0)	europea (100)	PCR	Entrevistas estructuradas a los pacientes y sus padres	4(12,1)
5	Chua G *et al.*	423: 254(60)M; 169(40)F	10.8 +/- 5,42(Korea del Sur) 12.9 +/- 5,5(Hong Kong) 6.6+/-5,0(Wuhan)	asiatica (100)	PCR	Análisis de historias clínicas y entrevistas con los médicos	14(3,3)
6	Qiu C *et al.*	27 (no diferenciaron sexos)	16,6 +/- 0,7	asiatica y europea (no se determinó el % por etnia)	PCR	Análisis de las historias clínicas y entrevistas estructuradas	7(26,9)
7	King J *et al*.	1987: 989(49,8)M; 998(50,2)F	9,3 +/- 5,2	anglosajona (100)	PCR	Análisis de las historias clínicas y entrevistas estandarizadas	153(7,7)

Fuente: Elaboración propia

Al observar las tablas 3 y 4, encontramos que los 7 estudios fueron publicados en el 2020, las poblaciones observadas fueron de Europa (Croacia, Portugal y España), 1 en Rusia, uno en Canadá y 2 en ciudades en simultáneo (Corea, Wuhan y Hong Kong; China, Francia y Alemania). Los estudios seleccionados trabajaron exclusivamente con poblaciones de niños, excepto el publicado por Qiu C *et al.*^26^, quienes observaron la variable en adultos y niños. La muestra más grande corresponde a Gorelov A *et al*. ^22^ con n = 7469 niños diagnosticados con COVID-19 y que tenían como condición el ser pacientes sintomáticos. El estudio de King J *et al*. presenta la segunda muestra más amplia con 1987 niños que fueron diagnosticados con COVID-19 y que eran asintomáticos y sintomáticos. El estudio de Chua G *et al*. ^25^ es el tercero con la muestra más amplia, con 423 niños de las ciudades de Corea (n = 91), Wuhan (n = 244) y Hong Kong (n .= 88), se incluyeron sintomáticos y asintomáticos. Los otros 4 estudios, Krajcar N *et al*. ^21^ (n = 151), Picao de Carvalho C *et al.*^23^ (n = 103), Ozores A *et al*. ^24^ (n = 33) y Qiu C *et al.*^26^ (n = 27) trabajaron con muestras pequeñas para su población, e incluyeron asintomáticos y sintomáticos. 

## DISCUSIÓN

El gusto en el ser humano se relaciona con la identificación de compuestos que provocan las sensaciones de dulce, salado, amargo, agrio y umami ^28^. Las alteraciones quimiosensoriales en el sistema gustativo producen cambios del gusto cuantitativos (ageusia e hipogeusia) y cualitativos (disgeusia) ^29^. Con respecto a la definición de trastornos del gusto en esta revisión, los términos usados por los trabajos fueron de disgeusia, en Picao de Carvalho *et al*. ^23^, Ozores A *et al.*^24^; ageusia, en Krajcar N *et al*. ^21^, Gorelov A *et al*. ^22^, Chua G *et al*. ^25^ y King J *et al*. ^27^; y disfunción gustativa, en Qiu C *et al*. ^26^. 

Para el diagnóstico de los trastornos del gusto, normalmente se utilizan soluciones líquidas para estudiar las cuatro cualidades: dulce, agrio, salado y amargo ^30^^,^^31^. Las soluciones están típicamente hechas de sacarosa, ácido cítrico, cloruro de sodio y clorhidrato de quinina o cafeína ^(32, 33)^. Otros medios para probar la función gustativa psicofísicamente son pruebas de boca completa con líquidos, comprimidos o tiras gustativas, conocidas como pruebas del umbral de reconocimiento, pero también se puede diagnosticar mediante la anamnesis al paciente ^34^^-^^36^. 

En los artículos revisados se diagnosticaron los trastornos del gusto con base en la anamnesis realizada a los pacientes y registrada en sus historias clínicas. Además, Chua G *et al*. ^25^ consultaron con los médicos tratantes para completar información relevante, Krajcar N *et al*. (21), Picao de Carvalho C *et al*. ^23^ y Ozores A *et al.*^24^ consultaron con los padres y tutores para profundizar en la sintomatología. Por su parte, Qiu C *et al.*^26^ realizaron entrevistas estructuradas con el apoyo de la escala de valoración analógica (EVA) para la valoración del gusto ^37^. Por tanto, la evaluación del sentido del gusto, en su mayoría, ha sido subjetiva e indirecta, por lo que se requiere investigaciones que valoren los trastornos del gusto con pruebas de mayor rigor, como las pruebas sensoriales del umbral de reconocimiento.

En cuanto a la prevalencia de los trastornos del gusto encontramos lo siguiente: Krajcar N *et al*. ^21^ trabajaron en una muestra que correspondía a dos periodos de tiempo de Croacia, conocidos como la primera y segunda ola. Contaron con una muestra total de 230 personas de la que extrajeron solo a los pacientes entre 7 y 18 años, que sumaban un total de 151; de estos, el 17,9% presentaron anosmia y ageusia. 

Gorelov A *et al*. ^22^ llevaron a cabo su estudio entre enero y junio del 2020, periodo durante el cual Rusia registró 47 712 casos confirmados de COVID-19 en niños, lo que representaba el 8,4% del total de infectados en la nación. El estudio accedió a 19 176 historias clínicas, de las cuales se reportó que el 32,3% (6203) son pacientes asintomáticos y el 67,7% (12973) sí presentó uno o más síntomas. Se tomaron en cuenta 7469 historias clínicas de pacientes que reportaron síntomas en la fase inicial de la enfermedad (rinitis, rinofaringitis, fiebre vómitos, nauseas diarreas) y se halló que el 17,1% (1277) presentaba anosmia/ageusia.

Picao de Carvalho C *et al*. ^23^ trabajaron con 103 historias clínicas de pacientes menores de 18 años registrados en la Plataforma COVID, entre el 11 de marzo y el 18 de junio del 2020; de ellos solo el 10% presentaba un factor de riesgo (nacimiento prematuro, dolencia metabólica, asma, diabetes y trasplante renal). El 80% fueron pacientes sintomáticos y el 20%, asintomáticos. Entre los principales signos y síntomas se encontraron tos, rinorrea, odinofagia, hipoxemia, náuseas, vómitos, diarrea, cefalea, mialgias y artralgias. En cuanto a las manifestaciones clínicas sensoriales, se registró anosmia (1%) y ageusia (4%). 

Para Ozores A *et al*. ^24^, la población de referencia fue de 73 000 niños entre 0 y 15 años, durante el periodo de marzo a junio del 2020, de los cuales 1650 fueron positivos para la infección por coronavirus. El estudio seleccionó 126 niños, de los cuales en el 2,2% ^33^ se confirmó su infección por coronavirus y 93 se descartaron. Entre los pacientes sintomáticos se halló al menos una de las siguientes manifestaciones clínicas: fiebre, dificultad respiratoria, anosmia/hiposmia, mialgias, dolor torácico, diarrea, odinofagia, cefalea, lesiones cutáneas compatibles con vasculitis, ageusia/disgeusia y rinorrea. La frecuencia de la disgeusia como signo clínico inicial ha sido del 3% y, a lo largo de todo el proceso, del 12,1%. 

Chua G *et al*. ^25^ trabajaron en una población estratificada en tres ciudades (Corea, Hong Kong y Wuhan), con una muestra total de 423 niños y adolescentes entre 0 y 18 años; 111 fueron asintomáticos (20 de Corea del Sur, 40 de Hong Kong y 51 de Wuhan) y 312 (71 de Corea del Sur, 48 de Hong Kong y 193 de Wuhan) sí tuvieron manifestaciones clínicas (fiebre, mialgia, rinorrea, congestión nasal, ageusia, anosmia, diarrea, vómitos). La ageusia se manifestó en el 3,3% de los pacientes, de los cuales el 1,93% son de Corea y el 1,45% de Hong Kong; sobre Wuhan no hubo información disponible al respecto. Dos de las personas afectadas por la ageusia se ubicaron en el rango de 5-12 años y doce, en el rango de 12-18 años. Qiu C *et al.*^26^ realizaron el estudio con una muestra que incluía adultos, la parte de la muestra que correspondió a niños sumaba 27, de los cuales el 26,9% presentó trastornos del gusto. El estudio de King J *et al*. ^27^ se llevó a cabo entre el 8 de abril y el 5 de octubre del 2020, en pacientes menores de 18 años que fueron ingresados al Informe Salud Pública de Alberta. De un total de 3474 historias, fueron seleccionados los positivos a la infección por coronavirus, y quedaron al final 1987 niños. Los pacientes asintomáticos fueron el 35,9% (714) y los sintomáticos, el 59,1% (1273), y las manifestaciones clínicas encontradas en esta muestra fueron náuseas, vómitos, cefalea, anorexia, estornudos, fiebre, escalofríos, dolor muscular, mialgia, congestión nasal, malestar general, fatiga, disnea, dolor de garganta, diarrea, dolor de pecho, anosmia y ageusia. El 7,7% del total de niños y adolescentes positivos a la infección por coronavirus presentaron ageusia.

Tras el análisis, se encontró que la prevalencia estimada de los trastornos del gusto en la presente revisión sistemática osciló entre el 3,3% y el 26,9%, lo cual, a pesar de ser un rango amplio, difiere del estudio de Ranabothu S *et al*. ^38^, quienes encontraron pérdida del gusto en un 2,1% de la muestra de un total de 1353 niños y adolescentes entre 0 y 18 años, y de los resultados de Erdede O *et al.*^39^, que identificaron un 0,68% de pérdida del gusto en una muestra de 145 niños y adolescentes. De igual forma, los resultados difieren del estudio de Kaye *et al*. ^40^, quienes trabajaron con 237 encuestados, de los cuales el 2% eran niños o adolescentes y 3 de ellos presentaron pérdida del gusto.

Los resultados encontrados en esta revisión sistemática no se pueden generalizar, debido al bajo número de estudios y algunas muestras que carecen de representatividad; sin embargo, la presente revisión es un aporte inicial de la sistematización de los resultados sobre una variable poco estudiada aún en la población de niños y adolescentes. 

## CONCLUSIONES

Los trastornos del gusto son parte de las manifestaciones clínicas que se presentan en los pacientes con infección por coronavirus. En el presente estudio se ha reportado como pérdida total (ageusia), pérdida parcial (hipogeusia) o distorsión del gusto (disgeusia), y la prevalencia en niños y adolescentes osciló entre el 3,3% y el 26,9%. 

## Fuente de financiamiento:

Autofinanciado
